# Household factors and prevalence of squalor: meta-analysis and meta-regression

**DOI:** 10.1186/s12889-024-17983-3

**Published:** 2024-02-15

**Authors:** Mike Norton, Stephen Kellett, Vyv Huddy, Melanie Simmonds-Buckley

**Affiliations:** 1 University of Sheffield, Sheffield, UK; 2 Rotherham Doncaster and South Humber NHS Trust, Rotherham, UK; 3 Department of Psychology, University of Sheffield, Sheffield, UK; 4 Clinical and Applied Psychology Unit, Department of Psychology, University of Sheffield, Sheffield, UK

**Keywords:** Squalor, Diogenes Syndrome, Severe Domestic Squalor, Self-neglect

## Abstract

**Background:**

Severe domestic squalor occurs when a person lives in a dwelling that is significantly unclean, disorganised and unhygienic. The limited previous research has primarily focused on the characteristics of those who live in squalor and the associated risk factors. Robust and reliable studies of squalor prevalence have not been conducted. This study sought to produce a reliable estimate of the point prevalence of squalor.

**Methods:**

Using data from 13-years of the English Housing Survey, *N* = 85,681 households were included in a prevalence meta-analysis. Squalor prevalence over time, subgroup analysis and logistic regression investigated the role played by household and community characteristics.

**Results:**

The point prevalence of squalor was estimated to be 0.85% and squalor was seen to decrease significantly over time. More significant community deprivation, a rented dwelling, lower income and high numbers of people in the home was associated with a greater risk of squalor.

**Conclusions:**

Squalor prevalence was higher than previous estimates and supports community care services in associated service planning. The results regarding household characteristics help to inform which households and individuals may be at a higher risk of living in squalid conditions.

**Supplementary Information:**

The online version contains supplementary material available at 10.1186/s12889-024-17983-3.

## Background

The person living in a consistently and significantly unclean and disorganised home is often referred to in research as suffering from Diogenes Syndrome (DS) [1–5] or they are said to be residing in Severe Domestic Squalor (SDS). Snowdon suggests that the term SDS should be used when *“…a person’s home is so unclean, messy and unhygienic that people of similar culture and background would consider extensive clearing and cleaning to be essential.”* [6]. Two additional conditions, Hoarding Disorder (HD) and Self-Neglect (SN), share a number of features with squalor. However, a key feature of HD is the compulsive need to acquire and retain objects, unlike squalor, where accumulation of items is commonly passive [7]. Similarly, there is overlap between squalor and SN. However, SN refers to all forms of neglect of the self, not just environmental neglect [8]. Therefore, it may include individuals who are living in clean households, but neglecting personal hygiene, diet, or medical interventions [9]. For the individual, squalor can lead to physical safety risks, difficulty accessing and receiving services and associated isolation [10] and a raft of associated physical health problems [11, 12]. Furthermore, squalor creates significant problems for the individual’s family and their neighbours [13, 14]. The evidence base for squalor is thin, as it tends to be too narrowly focused on adults over 65 years old [15–18], has small sample sizes, is over-reliant on cross-sectional methods [15, 18–20], rarely have a control group, uses case identification approaches that lack reliability and validity [15, 16, 18, 19] and creates data not pertinent to the focus of the study [19–22]. This list of methodological concerns clearly limits understanding and generalisability.

Squalor research has also been too heavily focused on the characteristics of the individual, such as their mental and physical health, their cognitive profile and their awareness of their condition [23–25] and lacks information on the context in which the person lives. Unlike related conditions, such as SN and HD, which have researched household and local factors such as deprivation, community profiles, risk of crime, social resources and household income [26–29], squalor has only considered rates of home ownership and living alone. The limited information that is available reports ownership rates between 39–59% [10, 17, 18, 21, 30], although one study reported a much lower rate of 5% [23]. In addition, individuals living in squalor are the sole member of the household in approximately 65–94% of cases [3, 10, 15–17, 19, 20, 22, 23, 30], with Ito et al. [3] showing that lone living was significantly higher than in a non-squalor control group. The SN literature (I.e. [31, 32]), which includes, but is not limited to, people who live in squalor, contains a more comprehensive evidence base with regards household and context factors. Studies found that SN was linked to higher levels of deprivation [26, 33]. Furthermore, income and SN have also been shown to be related, with SN more common when income is lower [27, 34–36], though other SN studies contradict these findings [37, 38]. Living alone was identified as being significantly more common in individuals who SN [27, 39, 40] and this was also found in the related condition of HD [28, 41, 42].

An improved understanding of the local and household risk factors for squalor would support community services in identifying locations, dwellings and families that have an increased chance of deteriorating into squalid living. However, to effectively identify and support individuals in these circumstances requires an accurate understanding of the scale of the problem and reliable case identification. Unfortunately, the literature is lacking a reliable estimate of the prevalence of squalor due to poor case identification methods. The point, period and lifetime prevalence of squalor is therefore unknown. A different, but related estimate is the 'incidence rate’ which, like period prevalence, considers squalor cases over time, but only includes new cases [43]. Incidence rates for squalor have been calculated [17, 18, 23, 44] and estimates range from 0.05–0.12% in adults over 60, or 65 years. Only one study considered the occurrence of squalor across all ages [23], reporting an incidence rate of 0.03%. However, these studies had high risk of bias as they calculated incidence, not prevalence, with estimates drawn as a ratio from the number of referred cases per year from a known population size. Therefore, as stated by Snowdon and Halliday [17], true prevalence estimates would likely be “substantially higher”.

The present study will provide the first point estimate of squalor based on adults across all ages and furthermore, will base its estimate on a sample in which all types of dwelling are included, not relying on referred suspected cases. Case identification will be robust as this will be based on the valid and reliable methods used by the English Housing Survey (EHS) in which domiciliary visits form part of the robust assessment of the home environment. Also, in using data from multiple years of the EHS this will provide a large random sample from the general population (i.e., not just referred cases) and in adults across all ages (i.e., not just the over 65s). The EHS collects data annually from a random sample of households in England. However, the data does not use the same participants each year and is therefore not truly longitudinal, but does allow an estimate of the point prevalence of squalor year-on-year using a panel study approach [45]. No previous research has investigated how squalor levels have changed over time. Therefore, this study’s consideration of squalor prevalence in a series of annual datasets allows identification of possible temporal trends to be considered for the first time.

Prevalence meta-analyses combine estimates from multiple studies to produce a summary estimate of the rate of a disorder or occurrence [46]. In this study, the meta-analysis will synthesise results from 13 annual administrations of the EHS to produce a pooled estimate of the prevalence of squalor. This is novel in the squalor evidence base. Prevalence meta-analyses have become significantly more common in the last decade as they increase precision by minimising the error in the estimates [47]. By using this method, an estimate of the point prevalence of squalor can be produced that is more reliable and robust than previous estimates, with reduced heterogeneity due to the same method of case identification being used each year. A more reliable estimate will allow health and social services to effectively plan for the needs of individuals living in the community whose dwellings show signs of squalor [48]. Furthermore, by using a meta-analytical approach with subgroup analysis, it will be possible to identify the characteristics of households that have an increased risk of their dwelling becoming squalid. This will further inform services regarding where their resources should be focused to provide support to those most in need.

## Aims and Hypotheses

The aims of this study were as follows: Firstly, to estimate the point prevalence of squalor in the general population. This would be the first measurement of its kind, improving on previous calculations of squalor incidence by identifying cases from a large, reliable dataset of households over a significant timeframe, rather than producing an estimate based on the rate of new referrals. Secondly, the study aims to identify variability of the presence of squalor in the general population over time. This will be the first investigation of whether squalor prevalence is consistent or varies over a substantial time period, identifying temporal trends and informing future research into squalor in the general population. Finally, this study aims to investigate the relationship between squalor and household factors. Household and local factors have been investigated in previous studies of squalor and related conditions. However, the research has limitations. Nonetheless, the findings on deprivation, income and household characteristics suggest that they may act as risk factors for squalor. Therefore, two hypotheses focus on the role of household factors: (1) risk of household squalor will be higher in areas of more severe deprivation and when the household income is lower and (2) the size of the household, whether the home is owned or rented, and whether the individual lives alone will all predict squalor.

## Methods

### Source data

The current study used data provided by the EHS [49]. The EHS is a continuous national UK survey, first conducted in 1967, that collects information about people’s housing circumstances. The UK Statistics Authority states that the statistics in the EHS are “produced according to sound methods and managed impartially and objectively in the public interest.” [50]. Each year a sample of houses are drawn at random and invited to participate. Those that agree take part in a face-to-face interview survey and are invited to take part in the physical survey, where a qualified surveyor comes to the property and completes a visual inspection of the interior, exterior and local area. Around 13,000 households take part in the face-to-face survey and another 6,000 also allow their property to be subject to the physical inspection. This study will include data from the 2007/08 wave of the study, through to the 2019/20 version and so represents data from 13 separate years [51–57].

### Measures

The EHS collects data on a significant number of areas and topics. Data on each household is collected from an interview with an individual who lives there. Although the household may have multiple occupants, each residency is included only once, as the study investigates household, rather than individual, characteristics. Several variables from the EHS are included in this research.

#### Presence of squalor

This is based on a measure from the physical survey of the property. The surveyor rated the risk due to ‘domestic hygiene, pests and refuse’ inside the property. Potential ratings are ‘significantly lower risk than average’, ‘average’, ‘significantly higher risk than average’ and ‘extreme’. For the purposes of this study, any individual deemed to be at ‘significantly higher risk’ or ‘extreme’ risk were considered to be living in squalor.

#### Year

Data is analysed across 13-years of the EHS. The first instance of a question being asked about household cleanliness was in 2007/08. The same question was then asked every year, up to 2019/20. Recent data were not available, as physical inspections stopped due to the Coronavirus pandemic.

#### Local deprivation

Each area in which individuals were surveyed was given a deprivation score, with values from 1–10 identifying whether the area was in the most deprived 10% of areas, to the least deprived 10% of areas, respectively. The deprivation for each year group was based on data from the Index of Multiple Deprivation (IMD; [58]). The IMD is the official measure of relative deprivation in England. It is made up of seven distinct domains, including income, employment, education, health and disability, crime, housing and living environment, which are then combined and weighted [59]. Due to the low occurrence of squalor in some of the deprivation categories, it was not always appropriate to complete statistical analysis with deprivation separated into ten groups. Therefore, in these instances, the deprivation category was split into three groups: most deprived (Categories 1–3), average deprivation (4–7) and least deprived (8–10).

#### Gross household income

Total annual income from both the individual and their partner, including savings. Values from £0 to £100,000. Values of more than £100,000 are still given a value of £100,000. To enable a variety of statistical analyses to be completed, the continuous income data was also split into four quartiles to allow for analysis as a categorical variable.

#### Tenure

The ownership status of the house. Potential responses are owner occupier, private rented, local authority (LA) or housing association (HA). LA housing is provided by the local council, whereas housing associations are private, not-for-profit companies providing mainly low-rent housing [60]. Where a binary category was required for analysis and for comparison with previous research, this category was simplified to those who owned their home and those who did not.

#### Household type and size

These variables give information as to who is present in the household. 6 categories are included: couple with or without dependent children, lone parent, other multi-person household, one person (Under 60 years old) or one person (60 years and older). Dependent children are those that are part of the main family unit, who are under the age of 16, or between 16–18 and in full-time education. ‘Couple with children’ and ‘Lone parent’ both include dependent children. ‘Other multi-person household’ may also include dependent children in some cases [61]. Where a binary category was required for analysis and for comparison with previous research, this category was simplified to those who live alone and those who live with others. Household size reported the total number of individuals in the household, including children.

### Analysis strategy

Data from 13 years of the EHS were assessed and each separate year was treated as an independent data set. Therefore, analysis assessed 13 sets of EHS results. Analysis was conducted using the statistical programme R version 4.3.0. Initially, a meta-analysis and forest plot were completed, calculating a random effects model estimate of squalor prevalence and measures of heterogeneity. As the identified proportions were all close to 0, meta-analyses throughout the study were run using a Freeman-Tukey double arcsine transformation, to stabilise the variance [46, 62]. Analysis was then split into two areas. Firstly, the role of the variables and their influence on squalor, and secondly, their effect over time. For the first stage, subgroup analysis using individual participant meta-analysis, as described by Tierney et al. [63], was conducted to compare households on each independent variable. Yearly datasets were split into subgroups for each variable (deprivation, home ownership, whether they lived alone, income and household size). Then, meta-analyses for each level of the variable were completed and their prevalence rates compared. In addition, variables were also investigated with the complete dataset using logistic regression. As the squalor prevalence values are low, Firth’s Bias-Reduced logistic regression [64] was used to account for the rarity of squalor events in the datasets [65]. To analyse squalor prevalence over time, fixed prevalence data was calculated for each variable and for each year and displayed in values and as line graphs. Meta-regression was used to investigate whether squalor prevalence showed a significant change over time by isolating the data for each variable and each level and assessing whether there was a relationship between squalor prevalence and the year.

## Results

Table 1 summarises the complete dataset. Overall, *n* = 85,681 households were physically surveyed, with *n* = 763 identified as living in squalor, producing a fixed rate of 0.89%. Households that were squalid had on average 2.4 inhabitants and were more likely to be rented than owned by the resident.

**Table 1:** Summary of Data Set and Variables

Variable	Representative value
Total sample	85,681
Total squalor	0.89%
Average deprivation score	4.966 (2.904)
Average household income	24,886 (17,875)
Average household size	2.386 (1.336)
Tenure	
Owner occupied	45.87%
Not owned	54.13%
Private rented	18.85%
Local Authority housing	16.05%
Housing Association housing	19.23%
Owned occupied	45.87%
Not owned	54.13%
Household type	
Couple with no children	31.36%
Couple with children	21.42%
Lone parent	9.90%
Other multi-person household	8.04%
One person less than 60	12.41%
One person 60 +	16.87%
Living alone	29.28%
Living with others	70.72%

Average scores refer to Mean. Standard Deviation in brackets

**Fig. 1:**
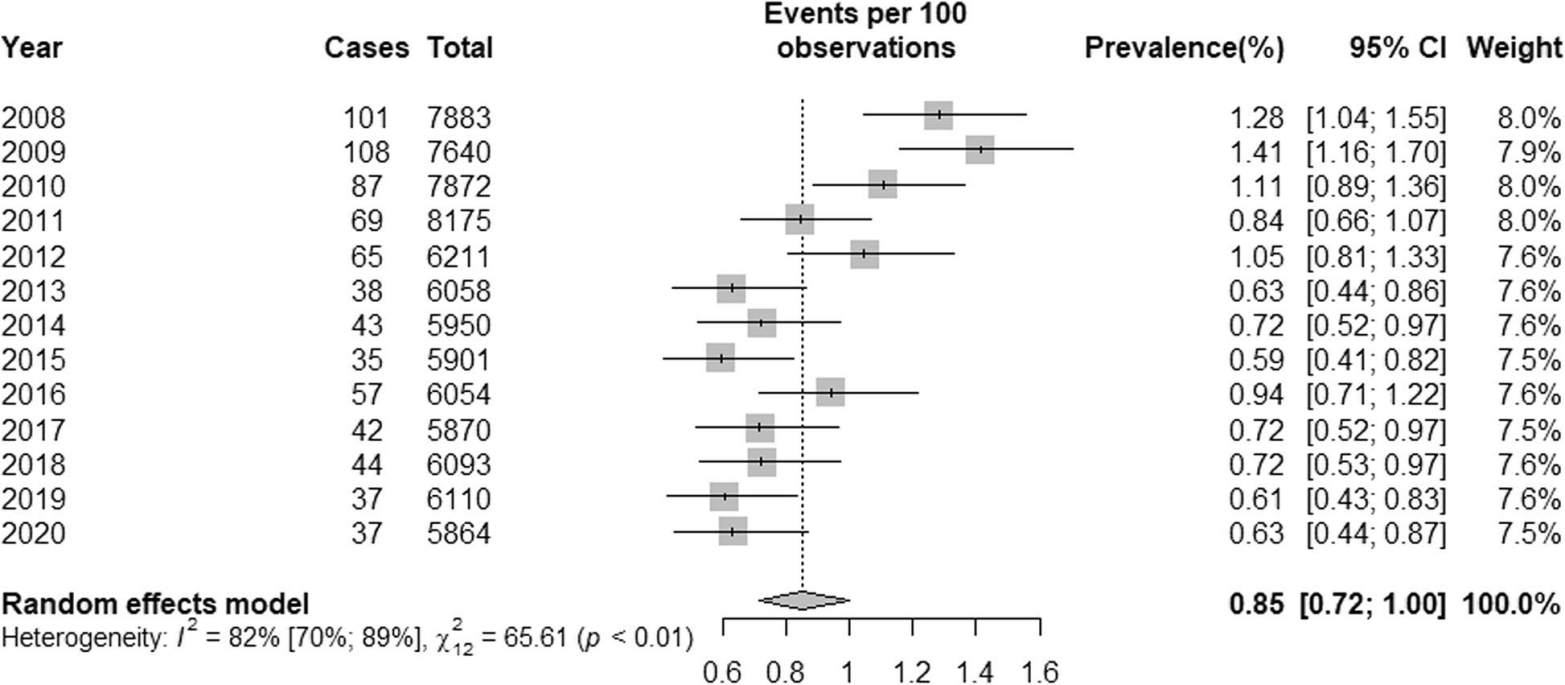
Forest Plot of Squalor Prevalence by Year

Figure 1 shows the results of the meta-analysis. The 13 yearly datasets totalled *n* = 85,681 households, producing a point prevalence estimate of squalor of 0.85% (95% CI’s 0.72 to 1.00). There was a significant and high heterogeneity between years of the EHS (*I*^*2*^ = 82%, 95% CI 70% to 89%, *Q* = 65.61, *p* < 0.01).

Table 2 contains the subgroup analysis results and suggests that all the independent variables, except household type (*Q* = 1.54, *p* = 0.2151), had a significant effect on the presence of squalor. Squalor prevalence was higher in areas with the most deprivation (*Q* = 46.32, *p* < 0.0001), in households with income in the lowest quartile (*Q* = 105.61, *p* < 0.0001) and in houses which were not owned by the resident (*Q* = 30.31, *p* < 0.0001). The number of individuals in the household was also shown to have a significant effect on squalor prevalence, with 2-person households showing the lowest prevalence and households with 5 or more individuals having the highest risk (*Q* = 25.61, *p* =  < 0.0001).

**Table 2:** Subgroup Analysis for Moderators of Squalor Prevalence

Variable	Subgroup	Prevalence	95% CI	*p*-value	*I*^*2*^	*Q*	*p*-value
Deprivation						46.32	**< 0.0001**
	Most deprived	1.23%	1.04–1.43%	0.0015	62.3%		
	Average deprivation	0.76%	0.59–0.94%	0.0001	69.2%		
	Least deprived	0.38%	0.25–0.53%	0.0025	60.4%		
Income						105.61	**< 0.0001**
	0–25%	1.31%	1.08–1.57%	0.0019	61.5%		
	25–50%	1.05%	0.92–1.19%	0.7414	0.0%		
	50–75%	0.68%	0.49–0.90%	0.0001	69.1%		
	75–100%	0.36%	0.28–0.45%	0.7041	0.0%		
Home ownership						30.31	**< 0.0001**
	Owned	0.50%	0.41–0.60%	0.0399	44.9%		
	Not owned	1.15%	0.93–1.39%	< 0.0001	82.9%		
Household type						1.54	0.2151
	Living alone	0.94%	0.82–1.06%	0.2653	17.7%		
	Living with others	0.82%	0.66–0.99%	< 0.0001	80.7%		
Household size						25.61	**< 0.0001**
	1 person	0.94%	0.81–1.07%	0.2653	17.7%		
	2 people	0.62%	0.52–0.73%	0.2636	17.8%		
	3 people	0.76%	0.53–1.03%	0.0012	63.0%		
	4 people	1.01%	0.76–1.29%	0.0273	47.9%		
	5 + people	1.36%	0.88–1.94%	< 0.0001	69.5%		

Table 3 demonstrates the regression values when the data was analysed as a single dataset. The only variable which was not found to be a significant predictor of squalor was whether the individual lived in the household alone (OR = 0.91, 95% CI [0.78, 1.06], *p* = 0.21). Deprivation was found to be a significant predictor, suggesting a decrease in the prevalence of squalor of around 13% for each deprivation increment (OR = 0.87, 95% CI [0.84, 0.89], *p* < 0.0001). This would represent a 72% decrease in rate of squalor from the most deprived to the least. Similarly, whether an individual owned the home predicted squalor, with a rented home being 127% more likely to be squalid (OR = 2.27, 95% CI [1.94, 2.67], *p* < 0.0001). Income and household size were also both significant predictors of whether a household was living in squalor. When all significant variables were combined to form a model for predicting squalor, significance remained in all cases.

**Table 3:** Logistic Regression of Independent Variables and Squalor with the Complete Dataset

Variable	Coefficient	Standard Error	Odds Ratio	95% CI	*p*-value
Variables analysed separately					
Deprivation (1–10)	-0.1422	0.0137	0.8674	0.8442, 0.8909	** < 0.0001**
Income (£0–100,000)	-0.0000	0.0000	0.9999	0.9999, 0.9999	** < 0.0001**
Household Size	0.1303	0.0813	1.1392	1.0847, 1.1951	** < 0.0001**
Living alone/with others	-0.0989	0.0784	0.9058	0.7779, 1.0577	0.2095
Home owned/rented	0.8212	0.0815	2.2732	1.9411, 2.6723	** < 0.0001**
Combined model					
Deprivation	-0.0633	0.0149	0.9386	0.9111, 0.9671	** < 0.0001**
Income	-0.0000	0.0000	0.9999	0.9999, 0.9999	** < 0.0001**
Household size	0.2714	0.0247	1.3117	1.2486, 1.3782	**0.0024**
Home owned/rented	0.2199	0.0907	1.2459	1.0393, 1.4938	** < 0.0001**

**Fig. 2:**
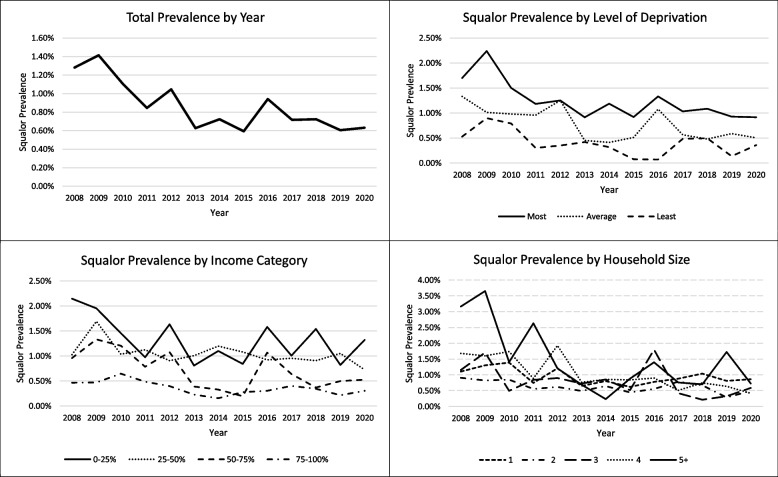
Squalor Prevalence Over Time and by Variable

Table 4 and Fig. 2 show the fixed prevalence rates and meta-regression results for each year and for each variable category. Overall, squalor prevalence significantly decreased over time (β = -0.053, 95% CI [-0.078, -0.028], *p* < 0.0001), with each year producing a decrease in squalor of around 0.05 percentage points. However, the main decrease in rate of squalor appears to have taken place in the first half of the time period, with squalor rate staying broadly consistent from 2013 onwards. Decreases in squalor rate were also identified in many of the variable categories, including all levels of deprivation and individuals who own, or rent privately. Households with two or more people, including couples with and without children and other multi-person households also showed a significant decrease in squalor rates.. The relationship between squalor prevalence and income was mixed, with households in the 50–75% category being the only ones to see a decrease in squalor rate over time. Those living in LA and HA housing, lone parents and those living alone did not observe the same decrease in squalor. Older adults living alone had a fixed squalor prevalence of 0.77%, which is lower than the overall rate.

**Table 4:** Squalor Prevalence Values (%) and Meta-regression by Time

	Total	Sq. cases	2008	2009	2010	2011	2012	2013	2014	2015	2016	2017	2018	2019	2020	All years	β-coefficient (CI 95%)	*p*-value
Total			7883	7640	7872	8175	6211	6058	5950	5901	6054	5870	6093	6110	5864	85,681		
Squalor cases			101	108	87	69	65	38	43	35	57	42	44	37	37	763		
Total prevalence	85,681	763	1.28	1.41	1.11	0.84	1.05	0.63	0.72	0.59	0.94	0.72	0.72	0.61	0.63	0.89	-0.0533 (-0.0784, -0.0282)	** < 0.0001**
Deprivation																		
Most deprivation	32,393	411	1.70	2.24	1.50	1.18	1.25	0.92	1.19	0.92	1.33	1.03	1.09	0.93	0.92	1.27	-0.0638 (-0.0985, -0.0291)	**0.0003**
Average deprivation	32,335	261	1.33	1.01	0.98	0.96	1.25	0.45	0.41	0.51	1.08	0.57	0.48	0.59	0.51	0.81	-0.0552 (-0.0904, -0.0199)	**0.0021**
Least deprivation	20,933	90	0.53	0.90	0.79	0.30	0.35	0.42	0.32	0.08	0.07	0.48	0.49	0.14	0.36	0.43	-0.0348 (-0.0676, -0.0020)	**0.0378**
Household income																		
0–25%	21,421	299	2.15	1.95	1.46	0.97	1.63	0.81	1.10	0.84	1.58	1.01	1.54	0.82	1.32	1.40	-0.0600 (-0.1212, 0.0012)	0.0547
25–50%	21,419	227	1.03	1.69	1.03	1.12	0.91	1.01	1.20	1.08	0.92	0.95	0.91	1.05	0.72	1.06	-0.0279 (-0.0633, -0.0075)	0.1223
50–75%	21,422	158	0.96	1.33	1.20	0.78	1.08	0.39	0.33	0.20	1.07	0.64	0.37	0.50	0.52	0.74	-0.0564 (-0.01033, -0.0096)	**0.0182**
75–100%	21,419	79	0.47	0.47	0.65	0.49	0.40	0.23	0.16	0.27	0.30	0.40	0.34	0.21	0.30	0.37	-0.0183 (-0.0394, -0.0029)	0.0904
Tenure																		
Owner occupied	39,304	208	0.47	0.78	0.78	0.71	0.54	0.43	0.44	0.36	0.45	0.38	0.59	0.30	0.26	0.53	-0.0307 (-0.0477, -0.0137)	**0.0004**
Not owned	46,377	555	2.04	2.10	1.48	1.00	1.42	0.77	0.90	0.76	1.27	0.98	0.81	0.85	0.94	1.20	-0.0817 (-0.1288, -0.0347)	**0.0007**
Private rented	16,148	212	3.11	2.81	1.92	0.72	1.86	1.00	0.83	0.84	1.03	1.20	0.64	0.71	0.60	1.31	-0.1432 (-0.2204, -0.0661)	**0.0003**
Local Authority housing	13,756	186	1.92	2.31	1.80	1.47	1.00	0.82	1.04	0.52	1.54	0.62	1.01	0.93	2.03	1.35	-0.0705 (-0.1464, 0.0054)	0.0688
Housing Association housing	16,473	157	1.30	1.24	0.73	0.94	1.33	0.50	0.86	0.88	1.27	1.01	0.83	0.93	0.50	0.95	-0.0287 (-0.0708, 0.0134)	0.1816
Household type																		
Couple with no children	26,870	148	0.61	1.03	0.69	0.55	0.70	0.28	0.57	0.45	0.49	0.59	0.37	0.22	0.38	0.55	-0.0354 (-0.0581, -0.0127)	**0.0022**
Couple with children	18,355	144	1.49	1.59	1.08	0.72	1.26	0.48	0.24	0.30	0.52	0.31	0.47	0.62	0.59	0.78	-0.0773 (-0.1279, -0.0267)	**0.0028**
Lone parent	8484	130	2.43	1.99	1.65	1.52	0.62	0.87	1.76	1.19	2.78	2.15	1.04	0.93	0.60	1.53	-0.0717 (-0.1604, 0.0171)	0.1136
Other multi-person household	6887	102	2.66	2.29	1.31	2.08	1.70	1.90	0.80	1.00	2.04	0.00	1.16	0.89	0.88	1.48	-0.1414 (-0.2432, -0.0396)	**0.0065**
One person < 60	10,629	127	1.99	1.60	2.17	0.62	1.52	0.48	1.06	0.61	1.18	1.15	0.68	1.07	0.93	1.19	-0.0625 (-0.1377, 0.0127)	0.1034
One person ≥ 60	14,456	112	0.55	1.04	0.79	0.84	0.93	0.80	0.60	0.63	0.49	0.69	1.28	0.64	0.81	0.77	0.0001 (-0.0358, 0.0361)	0.9947
Living alone	25,085	239	1.11	1.30	1.39	0.74	1.20	0.65	0.80	0.62	0.78	0.88	1.04	0.81	0.86	0.95	-0.0270 (-0.0607, 0.0067)	0.1163
Living with others	60,596	524	1.36	1.46	0.99	0.88	0.98	0.62	0.69	0.58	1.01	0.65	0.59	0.52	0.52	0.86	-0.0626 (-0.0873, -0.0378)	** < 0.0001**
Household size																		
1 person	25,085	239	1.11	1.30	1.39	0.74	1.20	0.65	0.80	0.62	0.78	0.88	1.04	0.81	0.86	0.95	-0.0270 (-0.0607, 0.0067)	0.1163
2 people	29,474	188	0.91	0.82	0.85	0.55	0.62	0.49	0.64	0.44	0.56	0.79	0.67	0.29	0.49	0.64	-0.0329 (-0.0559, -0.0099)	**0.0051**
3 people	13,477	113	1.15	1.70	0.50	0.84	0.90	0.73	0.86	0.52	1.80	0.42	0.21	0.33	0.59	0.84	-0.0619 (-0.1095, -0.0144)	**0.0107**
4 people	11,310	122	1.68	1.60	1.74	0.89	1.93	0.76	0.86	0.84	0.90	0.51	0.75	0.64	0.42	1.08	-0.0939 (-0.1402, -0.0475)	** < 0.0001**
5 + people	6335	101	3.16	3.65	1.40	2.63	1.21	0.69	0.23	0.88	1.40	0.76	0.70	1.72	0.73	1.59	-0.1517 (-0.2739, -0.0295)	**0.0150**

## Discussion

The squalor evidence base has previously lacked a reliable estimate of the prevalence of squalor and what significantly predicts living in squalid home. Squalor prevalence estimates have been unreliable as they used individuals already identified as living in squalor and have been mostly limited to older adult samples. The current panel study sought to correct these limitations by providing the first reliable estimate of the prevalence of squalor across all ages and over time. The present study therefore took a novel meta-analytic approach, studying a large dataset from a random sample of the general population and using a reliable assessment method. However, using the EHS as a source of data on squalor is limited by the data collection processes involved. All surveyed households voluntarily agreed to be included in the EHS. It is possible that those who refused access to their homes varied significantly from those that agreed, suggesting that the sample used in the present study may have been influenced by selection bias. Individuals living in squalor are socially withdrawn [66–68] and will refuse entry to their property or any form of help [69–71], which would drive avoidance of engagement with the EHS. It is not practical to expect a study to enforce entry into homes. Therefore, future studies should consider novel methods to account for the voluntary nature of the survey.

The aims of this study were to provide a reliable estimate of the prevalence of squalor in the general population, to investigate the relationships between squalor and household factors and to investigate annual trends in the prevalence of squalor. Although there were limitations with the sampling processes, the aims of the study were met, and the results can now be discussed and connected to the extant evidence base.

### Squalor prevalence

The meta-analysis conducted on the EHS data used each yearly dataset as a separate event, generated an estimated squalor prevalence of 0.85%. Previous estimates had been between 0.05–0.12% in older adults [17, 18, 23, 44] and 0.03% across all ages [23]. Therefore, the point prevalence rate suggested by the present study is higher than previous estimates and has been achieved using more reliable methods. Previous research has relied on producing squalor rate estimates based on extant referral rates. In each study, the number of cases referred to a service per year and the population of the area was used to calculate prevalence. The present study, which estimated prevalence from the results of surveying over 85,000 dwellings therefore used a more reliable and extensive dataset, acquired using robust in situ interviewing methods and actual home visits. In using a meta-analytical approach with a larger dataset, a more reliable and precise estimate of prevalence of squalor has been achieved [72].

### Household factors

Deprivation, when broken down into three categories from the most deprived to the least deprived areas, showed a significant relationship with squalor prevalence, with squalor being over three times more likely in the most deprived areas than in the least. This was supported by results of the regression analysis. Deprivation was a significant predictor of squalor, with reduced deprivation being associated with a 13% decrease in the risk of living in a squalor for each increment from 1 (Most deprived) to 10 (Least deprived). These findings support the first hypothesis and previous evidence regarding the role of deprivation in predicting whether an individual lives in squalor [30] and the SN evidence base which has previously identified a relationship with deprivation [26, 33]. This suggests that deprivation should now be considered as a risk factor for squalid living and that squalor is not evenly distributed throughout society, but rather is more likely in communities suffering high deprivation. This is in line with numerous other negative outcomes which are also associated with deprivation, including measures of health, child well-being and crime [73–76].

The first hypothesis investigated whether squalor would be more common in households with a lower income and the results found this to be the case. Income has not previously been considered as a main variable in the squalor literature. Households in the lowest quarter of income were more than three times more likely to be squalid than households in the highest income quartile. Income was also related to squalor prevalence in the logistic regression, even when included with other variables. Income appears therefore to be a new variable of interest in squalor evidence base. The findings regarding income do mirror many SN studies that have found poor income to be a risk factor [27, 34–36, 39, 77], whilst all of these studies have been limited to using older adult samples (60 + or 65 +). Therefore, the outcomes of the present study are novel in that they investigate squalor, but also, in finding income to be a factor when investigating all adults, not just older individuals. People on low incomes will not be able to employ the cleaning and repair services that would enable a significant change to the home environment.

The second hypothesis suggested that additional household factors, such as whether someone lives alone, home ownership and how many people reside in the dwelling would also predict the home being squalid. Both squalor and SN literature have previously reported on key household factors [3, 27, 36, 39, 40]. This current study demonstrated that home ownership and household size were related to squalor prevalence, both in the subgroup analysis and individual regression. Individuals who did not own their own homes had a squalor prevalence more than double that of homeowners. Furthermore, when ownership was included in the regression with income and deprivation, it was still a significant predictor of whether an individual lived in squalor, suggesting that it has an effect beyond other variables. This could potentially be because individuals who own their homes are more likely to look after the property, as they bear the full costs of any wear and tear [78]. However, it may also be related to wealth, with homeowners shown to have more non-housing wealth [79], which could be used to afford cleaners or provide more free time for home maintenance.

Squalor prevalence was shown to vary significantly by household size, with the individual regression suggesting that increased household size predicted increased risk of squalor. This may be explained by social loafing, whereby individuals in groups make less effort than when they are working individually [80]. However, it is worth noting that squalor did not increase linearly with household numbers. Prevalence calculations in the subgroup analysis showed that the lowest rate of squalor was seen in 2- and 3-person households and the highest in those with 4 or more individuals, with the prevalence in single-person households somewhere in between. Potentially, individuals living alone may struggle to find the time to maintain a household by themselves, or they may have less motivation to do so if no one else is regularly present. Further research in this area would be required to fully understand these findings.

Solitary living is one of the few household variables that has been covered in multiple squalor studies and has also received attention in related disorders, such as SN and HD. Rates of living alone in squalor cases have been shown to be high [3, 10, 15–17, 19, 20, 22, 23, 30]. Furthermore, Ito et al. [3] showed that, compared to a control group, individuals living in squalor were more likely to be living alone. Studies have also consistently found that individuals who SN or hoard are more likely to be living alone [27, 28, 39–42]. In the present study, the rate of squalor in individuals living alone was higher than multiple-person households. However, the difference was not significant at the 0.05 level. Furthermore, living alone also showed no relationship with squalor in the logistic regression. The lack of a relationship in the present study appears to disagree with the research base, as it suggests that squalor is not more common in individuals living alone. However, this may be due to the age of the participants in previous studies. The SN studies referenced [27, 39, 40] and the single squalor study which used a control [3] only investigated older adults. Therefore, this may suggest that if the EHS data was limited to older adults, they may have identified significantly higher rates of squalor in individuals living alone. However, the rate of squalor for the over 60’s living alone in the sample is lower than all but one other category (Couple with no children), suggesting this may not be the case.Further studies on squalor in the general population would need to be completed to clarify the findings. Additional research could also build on the findings of this study by introducing more in-depth analysis of the variables that have been shown to be risk factors. This could consider whether all factors contribute to an understanding of squalor risk, or whether some are primary predictors that should be the focus of future research and community identification processes.

### Time trends

Studies that assess squalor over time are extremely rare and are usually conducted unsystematically. Furthermore, they are limited to follow-up data assessing outcomes from identified clinical samples [3, 15, 17, 18, 44, 81] rather than people living in the community. Although this study was not truly longitudinal because it adopted a panel approach [45], it does represent a significant step in squalor research. This is because it analysed trends for the first time in reliable case identification of squalor prevalence taken over a 13-year period. This identified a significant relationship between squalor prevalence and time, such that squalor appeared to be decreasing over the 13-years. As the methods of the EHS have remained the same, then this decrease is unlikely to be due to measurement error or variation. However, during this study period, the EHS demonstrated an overall decrease in non-decent homes [82], so the decrease in squalid homes may be explained by an overall improvement in housing conditions. More specifically, the rate of squalor showed a notable decrease in the period from 2009–2013, with little improvement in more recent years (Fig. 2) and this trend is also seen in the rate of non-decent homes [82]. However, more detailed research into these measures would be required to identify whether this is a significant association.

A significant decrease in squalor prevalence was also observed in many of the variables and categories, such as homeowners, couples and multiple-person households. However, other groups did not show the same pattern. For instance, households renting privately saw a decrease in their rates of squalor, but this was not the case in LA and HA rented housing. Furthermore, both the lowest and highest income groups saw no significant decrease in squalor prevalence, whereas the lowest and highest deprivation groups did show a decrease over time. On the whole, groups that would be considered to be of a low income, such as the below average income groups (0–25% and 25–50%), LA and HA housing and lone parents, showed no significant decrease in squalor rates. However, further research into national patterns should be conducted to consider these findings in more detail.

### Strengths and Limitations

The strengths of this study are based around the use of the EHS as a dataset and the analytical methods employed. The EHS has been running annually for over 50 years and reports regularly on technical processes and data quality [83]. Therefore, the data included appears robust and reliable and the surveying methods were gold standard in that a domiciliary visit was conducted to identify squalid homes [84]. In addition, the significant size of the survey has allowed for a squalor sample to be produced from a general population, even when the prevalence of squalor was likely to be low. This makes the research unique, as no previous study has investigated squalor in a sample of this reliability and size. Secondly, regarding the analysis used in the study, no previous squalor research has been able to use a meta-analytical approach, as this has been the only study using multiple datasets. This has allowed for a robust random effects estimate of squalor prevalence.

There are limitations with the use of the EHS to collect data on squalor. Firstly, as previously discussed, it only includes those who agree to engage with the EHS and have their homes surveyed. Secondly, household conditions were measured on a 1–4 scale. This is not a validated measure, has not been used in previous squalor research, and little information is available regarding the conditions of a dwelling that would constitute inclusion into each category. Therefore, it is difficult to assess whether the individuals considered to be living in squalor in this study had similar living conditions to those identified in other squalor studies. Furthermore, those receiving a score of three, described as ‘significantly above average risk from domestic hygiene, pests and refuse’ could potentially be assigned that score due to a temporary lack of cleaning. Similarly, those who received a score of 2 (‘Average’) may normally live in conditions of mild squalor, but have made an effort to improve their dwelling due to the survey taking place. This is another issue related to the voluntary nature of the survey and the reliance on a single survey visit. In addition, out of over 85,000 households, only 763 were identified as living in some form of squalor. This prevalence of less than 1% makes for an unbalanced sample and restricted the use of some analytical procedures. Furthermore, it meant that when data was separated by year, certain categories, such as deprivation, could not remain in their original groupings, as the numbers of squalor cases were low or zero. By creating smaller groupings, some of the accuracy of the data was lost and patterns in the analysis more difficult to identify. A final limitation of the study is the lack of focus on the presence of children in the household. To allow comparisons with previous research, the data was separated into those who were living alone and those who were not. However, the presence of children in the dwelling could also have been considered as a risk factor. Post-hoc analysis (Supplementary material 1) offers some limited support that the presence of children increases the risk of squalor significantly. Further study in this area should consider this as an additional factor to be assessed.

### Practical implications

A better understanding of squalor is vital for those who work in the field. Compared to conditions such as Hoarding Disorder [85] and SN [86, 87], much less is known about individuals who choose to create and live in squalid homes and how they can be supported. A more reliable estimate of prevalence is an important step in supporting individuals living in squalor as it allows services to plan appropriately for the health care needs of these individuals [48]. There is an absence of empirically supported and evidence-based interventions for squalor. Evidence that squalor occurs in almost 1% of households enables workforce calculations and emphasises the importance of having professionals who are trained in how to psychologically manage the people and also change the environmental conditions. Interventions are likely multi-disciplinary, and the ratios of professionals and their time is currently unknown. This study has also helped inform the understanding of the local areas and types of households that are most at risk of living in squalor, including areas of significant deprivation, and low income, rented households where an individual lives alone, or where there are 4 or more residents. This can help direct resources into the locations where squalor is most likely, ensuring the available support is used most efficiently and effectively.

## Conclusions

This study used a unique approach in squalor prevalence research, investigating adults of all ages, taking from a general population survey and conducting a prevalence meta-analysis using 13-years of panel data. Furthermore, unlike many published squalor studies, it did not focus on the individual level demographics, but instead the household and local area context factors, which have received little previous attention. The study produced a squalor prevalence estimate higher than identified in previous squalor studies, suggesting that squalor could be more common than previously realised. The study showed a significant relationship between squalor prevalence and the variables of local deprivation, household income, home ownership and household numbers. However, no relationship was found when considering whether individuals lived alone, which contradicted previous squalor and SN research. Time trends, which have also received little attention in squalor, were also investigated, finding a significant decrease in squalor prevalence between 2008 and 2020. Robust case assessment methods, engagement strategies and multidisciplinary interventions packages now need to be developed and these interventions be thoroughly evaluated in well controlled outcome studies.

### Supplementary Information

**Supplementary material 1. **

## Data Availability

The datasets analysed during the current study are available in the UK Data Service repository, ukdataservice.ac.uk. The following datasets were analysed in this research: • 6612 – English Housing Survey 2008: Housing Stock Data • 7039 – English Housing Survey 2010: Housing Stock Data • 7511 – English Housing Survey 2012: Housing Stock Data • 8068 – English Housing Survey 2014: Housing Stock Data: Special Licence Access • 8387 – English Housing Survey 2016: Housing Stock Data: Special Licence Access • 8851 – English Housing Survey 2018: Housing Stock Data: Special Licence Access • 8922 – English Housing Survey 2019: Housing Stock Data: Special Licence Access Restrictions apply to the availability of some of these datasets, which were used under license for the current study, and so are not publicly available. Datasets 8068, 8387, 8851 and 8922 required completion of an application to access.

## Abbreviations

DS: Diogenes Syndrome
EHS: English Housing Survey
HD: Hoarding Disorder
IMD: Index of Multiple Deprivation
SDS: Severe Domestic Squalor
SN: Self-neglect
