# Supplementary material for: Household factors and prevalence of squalor: meta-analysis and meta-regression
Source: BMC Public Health. 2024 Feb 15;24:479. doi: 10.1186/s12889-024-17983-3 (PMC10870488; doi:10.1186/s12889-024-17983-3)
Supplement: Supplementary file 1 — Supplementary material 1. [file 12889_2024_17983_MOESM1_ESM.pdf]

Supplementary Material 1

Post-hoc analysis – Presence of children

Raw data

Dependent children	Total households	Squalor cases	Raw prevalence
Present	26839	274	1.02%
None	51955	387	0.74%

Subgroup analysis

Variable	Subgroup	Prevalence	95% CI	*p*-value	I^2	*Q*	*p*-value
Dependent children						4.56	.0328
	Present	0.96%	0.76-1.18%	.0001	69.0%		
	None	0.72%	0.62-0.84%	.0075	55.7%		
